# Melatonin protects vertebral endplate chondrocytes against apoptosis and calcification via the Sirt1‐autophagy pathway

**DOI:** 10.1111/jcmm.13903

**Published:** 2018-10-24

**Authors:** Zengjie Zhang, Jialiang Lin, Naifeng Tian, Yaosen Wu, Yifei Zhou, Chenggui Wang, Qingqing Wang, Haiming Jin, Tingting Chen, Majid Nisar, Gang Zheng, Tianzhen Xu, Weiyang Gao, Xiaolei Zhang, Xiangyang Wang

**Affiliations:** ^1^ Department of Orthopaedics The Second Affiliated Hospital and Yuying Children's Hospital of Wenzhou Medical University Wenzhou Zhejiang Province China; ^2^ Zhejiang Provincial Key Laboratory of Orthopaedics Wenzhou Zhejiang Province China; ^3^ The Second School of Medicine Wenzhou Medical University Wenzhou Zhejiang Province China; ^4^ The First Affiliated Hospital of Wenzhou Medical University Wenzhou Zhejiang Province China; ^5^ The Third Affiliated Hospital and Ruian People's Hospital of Wenzhou Medical University Ruian Zhejiang Province China; ^6^ Chinese Orthopaedic Regenerative Medicine Society Zhejiang University of School Medicne HangZhou China

**Keywords:** apoptosis, autophagy, calcification, intervertebral disc degeneration, melatonin, vertebral endplate chondrocytes

## Abstract

Melatonin is reportedly associated with intervertebral disc degeneration (IDD). Endplate cartilage is vitally important to intervertebral discs in physiological and pathological conditions. However, the effects and mechanism of melatonin on endplate chondrocytes (EPCs) are still unclear. Herein, we studied the effects of melatonin on EPC apoptosis and calcification and elucidated the underlying mechanism. Our study revealed that melatonin treatment decreases the incidence of apoptosis and inhibits EPC calcification in a dose‐dependent manner. We also found that melatonin upregulates Sirt1 expression and activity and promotes autophagy in EPCs. Autophagy inhibition by 3‐methyladenine reversed the protective effect of melatonin on apoptosis and calcification, while the Sirt1 inhibitor EX‐527 suppressed melatonin‐induced autophagy and the protective effects of melatonin against apoptosis and calcification, indicating that the beneficial effects of melatonin in EPCs are mediated through the Sirt1‐autophagy pathway. Furthermore, melatonin may ameliorate IDD in vivo in rats. Collectively, this study revealed that melatonin reduces EPC apoptosis and calcification and that the underlying mechanism may be related to Sirt1‐autophagy pathway regulation, which may help us better understand the association between melatonin and IDD.

## 1 INTRODUCTION

Low back pain (LBP) is a leading cause of disability and job‐related disability, affecting 80% of the population at some point during their life^1^ and may result in a low quality life and high healthcare costs.^2^, ^3^ Intervertebral disc degeneration (IDD) has been recognized as a major contributor to LBP^4^; however, IDD pathogenesis remains unclear. Intervertebral discs are composed of nucleus pulposus and annulus fibrosus, and the main function of intervertebral discs is to maintain the stability of the vertebrae. Above and below intervertebral discs, a layer of hydrated biological tissue exists, called the cartilaginous endplate (CEP). Since nucleus pulposus and annulus fibrosus tissues do not have their own vascular supply, the CEP is the main nutritional supply route for intervertebral discs.^5^ Loss of nutrient supply may lead to increased cell death, matrix degradation as well as decreased matrix production in intervertebral discs, which further lead to disc degeneration.^5^ Therefore, CEP dysfunction, which may block the nutritional supply for disc tissues, is believed to play a vital role in IDD pathogenesis.^6^ Excessive endplate chondrocyte (EPC) apoptosis and calcification are two major processes of cartilage endplate dysfunction.^7^

Sleep is an important process for humans: it allows the body to rest and restore physiological function, but sleep is associated with morbidity and mortality.^8^, ^9^ Sleep debt, which is increasingly prevalent in our current society,^10^ may disturb carbohydrate metabolism and endocrine function, contributing to age‐related chronic diseases.^11^ Circadian sleep disturbance has been reported to be tightly associated with various diseases, including obesity, diabetes, and cardiovascular disease.^12^ A prospective study in Finland showed that sleep disturbance is a risk factor for LBP development.^13^, ^14^ In addition, a 13‐year follow‐up study reported that sleep disturbance promotes LBP development.^15^ These studies imply that a correlation may exist between sleep and IDD.

Melatonin is an endocrine hormone synthesized and secreted by the pineal gland in the brain; it helps maintain circadian rhythms, such as sleep.^16^ Studies have shown the association between melatonin and IDD. Turgut's group reported that surgical pinealectomy may accelerate the IDD process in chickens.^17^ Their further studies revealed that exogenous melatonin administration activates the recovery process in degenerated disc tissue possibly by stimulating transforming growth factor β.^18^ Clinical studies have also demonstrated a positive correlation between the serum melatonin concentration and IDD in patients.^19^ Thereafter, melatonin receptors, including MT1 and MT2, were reported to be expressed in intervertebral disc tissues, and melatonin may downregulate MMP‐3/9 levels while upregulating COL2A1 and Aggrecan expression in disc cells.^20^ This evidence suggests that melatonin may play a vital role in the IDD process. Studies have reported that melatonin administration may reduce the cartilage endplate vascularity of degenerated intervertebral discs in rats.^21^ However, the mechanism of melatonin in IDD pathophysiology remains largely unknown, especially in EPCs.

In the current study, we applied tert‐butyl hydroperoxide (TBHP), which is commonly used as an exogenous inducer of oxidative stress,^22^, ^23^ to establish a pathological IDD condition in primary EPCs in vitro. We investigated the effects of melatonin on apoptosis and calcification in EPCs under oxidative stress and illustrated the possible mechanism of melatonin in ameliorating IDD processes.

## 2 MATERIALS AND METHODS

### 2.1 Ethics statement

All surgical interventions, treatments and post‐operative animal care procedures were performed in strict accordance with the Animal Care and Use Committee of Wenzhou Medical University (wydw2014‐0129).

### 2.2 Reagents and antibodies

Melatonin, 3‐methyladenine (3‐MA), TBHP and the type II collagenases were from Sigma‐Aldrich (St. Louis, MO, USA). The Sirt1, RUNX‐2, p62, β‐actin, OCN primary antibodies were acquired from Abcam (Cambridge, UK). The LC‐3, Beclin‐1, p‐p53, p53, p21, cleaved caspase‐3, Bax and Bcl‐2 antibodies were obtained from Cell Signaling Technology (Danvers, MA, USA). Alexa Fluor^®^ 488‐ and Alexa Fluor^®^ 594 labelled goat anti‐rabbit IgG (H+L) secondary antibodies were purchased from Jackson ImmunoResearch (West Grove, PA, USA). The 4′,6‐diamidino‐2‐phenylindole (DAPI) was obtained from Beyotime (Shanghai, China). The cell culture reagents were purchased from Gibco (Grand Island, NY, USA), the 0.1% Alcian Blue 8GX was from Sigma‐Aldrich, and the 6‐chloro‐2,3,4,9‐tetrahydro‐1*H*‐carbazole‐1‐carboxamide (EX‐527) was from Tocris Bioscience (Ellisville, MO, USA).

### 2.3 Surgical procedure

Thirty‐six Sprague–Dawley rats (200‐250 g) were randomly divided into three groups, including the control group (n = 12), IDD group (saline injected after surgery, n = 12) and melatonin group (melatonin injected after surgery, n = 12). As a previous study described,^24^, ^25^ the rats in the IDD and melatonin groups were anaesthetized by 2% (w/v) pentobarbital (40 mg/kg), and needles (27G) were used to puncture the whole annulus fibrosus layer though the tail skin. All needles were kept in the disc for 1 minute. After surgery, the melatonin solution (10 mg/mL) was immediately injected intraperitoneally to deliver a dose of 1 mg/kg/day in the melatonin group, and the IDD group was injected with saline every day until the rats were killed.

### 2.4 Primary rat EPC cell culture

More than ten Sprague–Dawley rats (5 male and 5 females, 100‐150 g) were killed with an overdose of sodium pentobarbital. Endplate cartilage was collected under a dissecting microscope. As a previous study described,^25^, ^26^ the tissues were digested by 2 mg/mL 0.1% type II collagenase for 4 hours at 37°C. Next, the digested cartilage tissues were incubated in DMEM (Gibco, Invitrogen, Grand Island, NY, USA) with 10% foetal bovine serum (FBS; HyClone, Thermo Scientific, Logan, UT, USA) and antibiotics (1% penicillin/streptomycin) and maintained with 5% CO_2_ at 37°C. The cells were harvested by using 0.25% trypsin‐EDTA (Gibco, Invitrogen) when cell confluent. Next, EPCs were passage into 10‐cm culture plates at the appropriate density. The complete medium was changed every other day and the first two and three passage EPCs were used in our experiments. The chondrocytes were cultured in an incubator maintained with 5% CO_2_ at 37°C. The complete medium was changed every other day.

### 2.5 Alcian blue and alizarin red staining

The chondrocyte phenotype was identified by staining for sulphated proteoglycans with alcian blue, as previously described.^26^ The EPCs were washed three times with phosphate‐buffered saline (PBS) and fixed with 4% paraformaldehyde for 15 minutes and with alizarin red solution (Genmed, Shanghai, China) for 30 minutes at 37°C. After washed by three times with PBS, the chondrocytes were pretreated with 0.1 M HCl for 3 minutes. Next, the cells were stained Alcian Blue solution for 30 minutes. After washed by distilled water for three times, the stained cells were observed, and images were captured with an inverted microscope (Nikon, Tokyo, Japan).

### 2.6 Cell viability assay

Melatonin cytotoxicity on EPCs was evaluated by the cell counting kit‐8 (CCK‐8; Dojindo Co, Kumamoto, Japan) assay according to the manufacturer's protocol. First, the EPCs were transferred to 96‐well plates (80 000 cell/cm^2^) and incubated with different melatonin concentrations (0, 0.5, 1, 2, 5 μM) for 24 hours. At the indicated time, the cells were washed with PBS, and then, 100 μL of DMEM containing 10 μL of CCK‐8 solution was added to each well of the plate and incubated for another 2 hours at 37°C. Then measured the OD at 470 nm using a micro‐plate reader. All experiments were performed triplicate.

### 2.7 Immunofluorescence

For immunofluorescence, as a previous study described,^23^, ^26^ EPCs were plated in six‐well glass plates, and then, after incubating with serum‐starved medium overnight, the cells were treated with 50 μM TBHP or were co‐treated with 50 μM TBHP and 1 μM melatonin for 2 hours in medium. The cells were washed three times with PBS before fixation using 4% paraformaldehyde, followed by permeation using 0.5% Triton X‐100 for 15 minutes. Then, cells were blocked with 10% bovine serum albumin for 1 hour at 37°C and incubated overnight at 4°C with the following primary antibodies diluted in PBS: Sirt1 (1:200), Runx‐2 (1:200) and LC3 (1:200). The next day, the glass plates were washed and incubated with Alexa Fluor^®^ 488/594‐conjugated secondary antibodies (1:400) for 1 hour at 37°C and labelled with DAPI for 5 minutes.

### 2.8 Western blot assay

Total protein was extracted from EPCs using RIPA with 1 mM phenylmethanesulfonyl fluoride (PMSF) and the protein concentration was measured using a BCA protein assay kit (Beyotime) according to manufacturer's protocol. The protein (30 μg) was separated with SDS‐PAGE and transferred to polyvinylidene difluoride membranes (Bio‐Rad, Hercules, CA, USA). After blocking with 5% non‐fat milk, the membranes were incubated with primary antibodies against Sirt1 (1:1000), cleaved caspase‐3 (1:1000), Bax (1:1000), Bcl‐2 (1:1000), Beclin‐1 (1:1000), LC3 (1:500), p62 (1:1000), P16INK4a (1:1000), Runx‐2 (1:1000), Osteocalcin (OCN; 1:1000), or β‐actin (1:1000) overnight at 4°C, followed by incubation with the respective secondary antibodies. As a previous study described,^25^ the bands were detected with electro‐chemiluminescence plus reagent (Invitrogen). Last, the band intensity was quantified with Image Lab 3.0 software (Bio‐Rad) by three observers who were blinded to the experimental groups.

### 2.9 TUNEL method

The terminal deoxynucleotidyl transferase dUTP nick‐end labelling (TUNEL) method has been recognized as a useful technique for measuring apoptotic DNA fragmentations. Cultured EPCs were harvested after 12 hours and were prepared in a six‐well plate. After fixing with freshly prepared 4% paraformaldehyde for 15 minutes, cells were incubated with 0.1% Triton X‐100 for 10 minutes at 4°C and washed thrice with PBS at every step. Cells were stained with an in situ cell death detection kit (F. Hoffmann‐La Roche Ltd., Basel, Switzerland) and DAPI according to the protocol. Apoptotic changes were observed, and image of apoptotic cells were preserved by a fluorescence microscope (Olympus, Tokyo, Japan).

### 2.10 Safranin o‐fast green staining

The rats were killed by an intraperitoneal overdose injection of 10% pentobarbital, and the tails were harvested at weeks 4 and 8 after surgery, according to the magnetic resonance imaging (MRI) changes in rats. The specimens were fixed in formaldehyde, decalcified, dehydrated and embedded in paraffin. The specimens were cut into 5‐μm thick sections. Sections of each disc were stained with safranin O (SO)‐fast green due to the protocol. The construction of intervertebral disc and the NP cells’ morphology and cellularity were observed by another three‐two‐blinded experienced histology researchers using a microscope (Leica, Wetzlar, Germany) and the histology score was evaluated according to a grading scale.

### 2.11 Magnetic resonance imaging method

After 4 or 8 weeks of surgery, the rats were given the MRI examination to evaluate the IDD. All rats were anaesthetized by an intraperitoneal injection of 10% pentobarbital (40 mg/kg). The rats were set up in prone position for MRI, and then the finger specific coil MRI mode was used for rats’ tail. As previous study described,^25^ Magnetic resonance imaging was performed on all rats to evaluate the signal and structural changes in sagittal T2‐weighted images using a 3.0 T clinical magnet (Philips Intera Achieva 3.0MR, Amsterdam, Netherlands). The degree of IDD was evaluated by Pfirrmann grading system.^27^

### 2.12 Sirt1 activity assay

The whole cell lysate was collected following treatment with RIPA buffer (Millipore, billerica, MA, USA) supplemented with the protease inhibitor PMSF. The total protein concentration of the samples was measured using a commercially available BCA protein assay kit (Spectra Max190; Molecular Devices, San Jose, CA, USA). Enzyme assays for SIRT1 activity were performed as per the manufacturer's instructions (Sigma‐Aldrich). The plates were read with a fluorescent spectrophotometer (Spectra Max190; Molecular Devices) at an excitation wavelength of 340 nm and emission wavelength of 430 nm.

### 2.13 Statistical analysis

Experiments were performed independently at least three times. The results are presented as the means ± SD. Statistical analyses were performed, using SPSS statistical software program 18.0. Data were analysed by one‐way ANOVA, followed by Tukey's test for comparison between control and treatment groups. Non‐parametric data (Pfirrmann grading) were analysed by the Kruskal–Wallis H test. Statistical significance was set at *P* < 0.05.

## 3 RESULTS

### 3.1 Melatonin treatment suppresses oxidative stress‐induced apoptosis and calcification in EPCs

**Figure 1:**
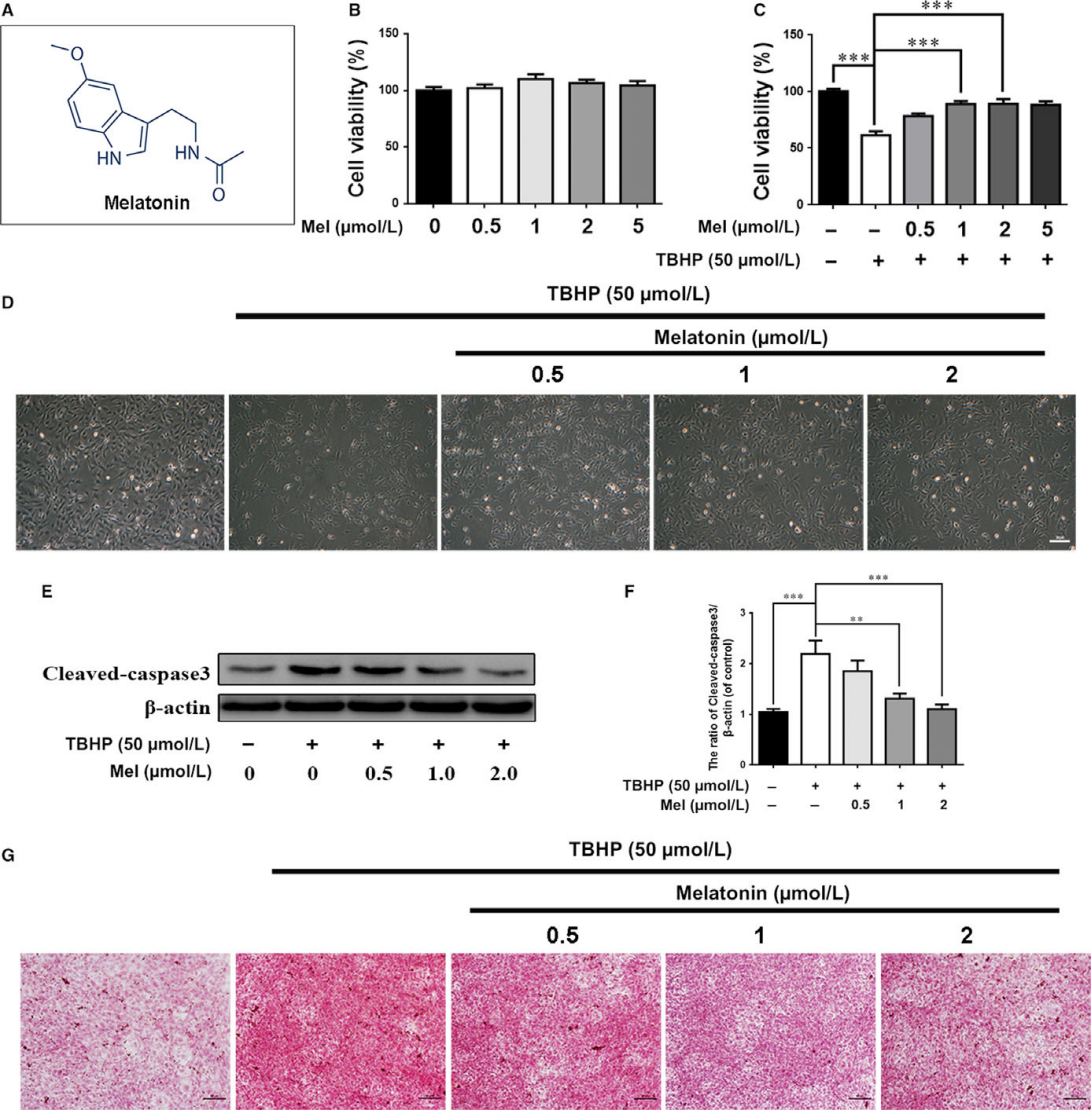
Effects of melatonin on cell viability, apoptosis and calcification of endplate chondrocytes. (A) Chemical structure of melatonin. (B) The cytotoxicity of melatonin on endplate chondrocytes was determined at various concentrations at duration of 24 hours using CCK8 assay. (C) Cells were treated with TBHP for 2 hours to induced oxidative stress, then cells were treated with melatonin for 4 hours, cell viability was measured by CCK8 assay. (D) Endplate chondrocytes were treated with melatonin (4 hours) and then TBHP (2 hours) and imaged by phase‐contrast microscopy (original magnification ×100, scale bar: 50 μm). (E) Protein content of cleaved caspase3 of endplate chondrocytes were evaluated by western blot. (F) Quantification of Cleaved‐caspase3 immunoblots. (G) Alizarin Red staining of cells. Endplate chondrocytes were treated with melatonin (4 hours) and then TBHP (2 hours), then cells were cultured in routine DMEM medium (without melatonin or TBHP) for 6 days and imaged by phase‐contrast microscopy (magnification ×100, scale bar: 50 μm). The experiment was repeated thrice, and a representative example is shown; the values presented are the means ± SD of three independent experiments. Significant differences between groups are indicated as ***P < 0.001, **P < 0.01 and ns *P* > 0.05

Before testing the effect of melatonin on EPC apoptosis and calcification, we evaluated the cytotoxicity of melatonin. The cytotoxic effect on chondrocytes was determined at various melatonin concentrations (0, 0.5, 1, 2, and 5 μM) over 24 hours using the CCK‐8 assay. As shown in Figure 1A, no marked melatonin cytotoxicity was noted in EPCs.

When cells were treated with TBHP, cell viability decreased, and melatonin induced substantial protective effects against TBHP‐induced cell death (Figure 1B and C; *P* < 0.01). After the TBHP treatment (50 μM), chondrocyte size was reduced, and some floating cells were observed, while melatonin markedly reversed this phenomenon (Figure 1D). In addition, the western blot results revealed that an apoptosis‐related protein (cleaved caspase‐3) was increased with TBHP treatment, but this effect was dose‐dependently reversed by melatonin treatment (Figure 1E and F; *P* < 0.01). Furthermore, the results from alizarin red staining, which is used to stain the calcified nodules produced by chondrocytes, revealed that calcification was markedly increased in chondrocytes with TBHP‐induced oxidative stressed, and melatonin partially ameliorated this calcification in chondrocytes.

These results suggest that melatonin protects chondrocytes against apoptosis and calcification under oxidative stress conditions.

### 3.2 Melatonin promotes autophagy in EPCs under normal and oxidative stress conditions

**Figure 2:**
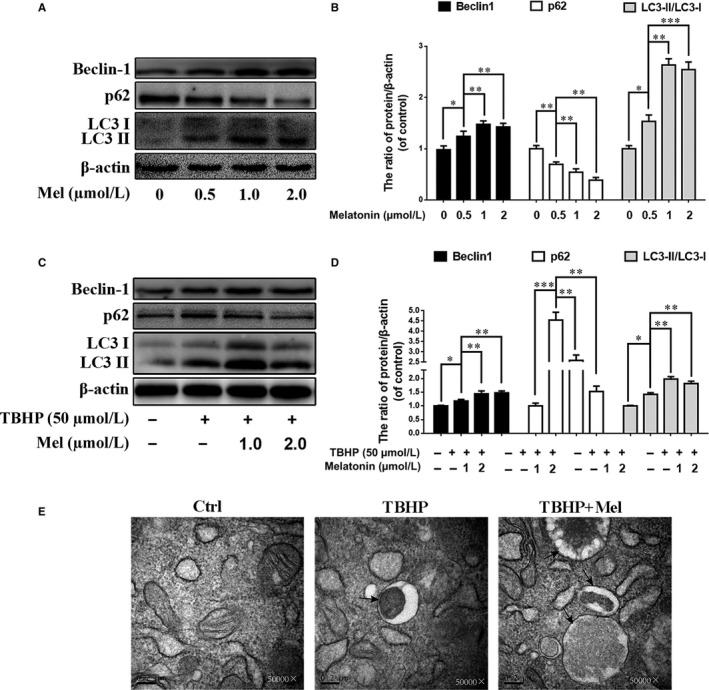
Melatonin treatment promotes autophagy in endplate chondrocytes. (A) Endplate chondrocytes were incubated with various concentration of melatonin for 24 hours. Protein content of Sirt1, LC3, Beclin‐1 and p62 of endplate chondrocytes were detected by western blot. (B) Quantification of immunoblots of Beclin1, p62, LC3‐II/I. (C) Endplate chondrocytes were incubated with various concentration of melatonin for 4 hours and then treated with TBHP (50 mM) for 2 hours. Protein content of LC3, Beclin‐1 and p62 of endplate chondrocytes were detected by western blot. (D) Quantification of immunoblots of Beclin1, p62, LC3‐II/I. (E) Morphology of autophagosomes was detected by transmission electron microscopy (×50 000) in endplate chondrocytes (Black arrow: autophagosome). The experiment was repeated at least three times, with a representative example shown; the values presented are the means ± SD of three independent experiments. Significant differences between groups are indicated as *** *P* < 0.001, **P < 0.01, *P < 0.05 and ns *P* > 0.05

Autophagy‐related proteins were detected by western blotting after treatment with various melatonin concentrations (0, 0.5, 1, 2, 5, 10 μM) for 4 hours. As shown in Figure 2A, the LC3‐II/LC3‐I ratio and Beclin‐1 expression, which are regarded as indicators of autophagosome formation, were substantially increased, especially in the 1 μM melatonin group (*P* < 0.01). Meanwhile, SQSTM1/p62 expression, which is an indicator of autophagic flux, was dose‐dependently decreased after the melatonin treatment.

We also found that TBHP slightly upregulated the LC3‐II/LC3‐I ratio, while this ratio was markedly increased after the melatonin treatment, peaking at 1 μM and decreasing at 2 μM (Figure 2C; *P* < 0.01). Melatonin also increased TBHP‐induced Beclin‐1 expression, and this effect was dose dependent. The expression of p62 was increased in the TBHP group while decreased in the TBHP and melatonin co‐treatment group. We used transmission electron microscopy to observe autophagosomes and autophagolysosomes. Compared with DMEM‐treated cells (control), autophagosomes and autophagolysosomes were observed in TBHP‐treated cells, and comparatively more autophagosomes and autophagolysosomes were found in the cells co‐treated with TBHP and melatonin (1 μM).

These data suggest that melatonin promotes autophagy in EPCs under normal and oxidative stress conditions.

### 3.3 Melatonin protects EPCs against apoptosis via autophagy stimulation

**Figure 3:**
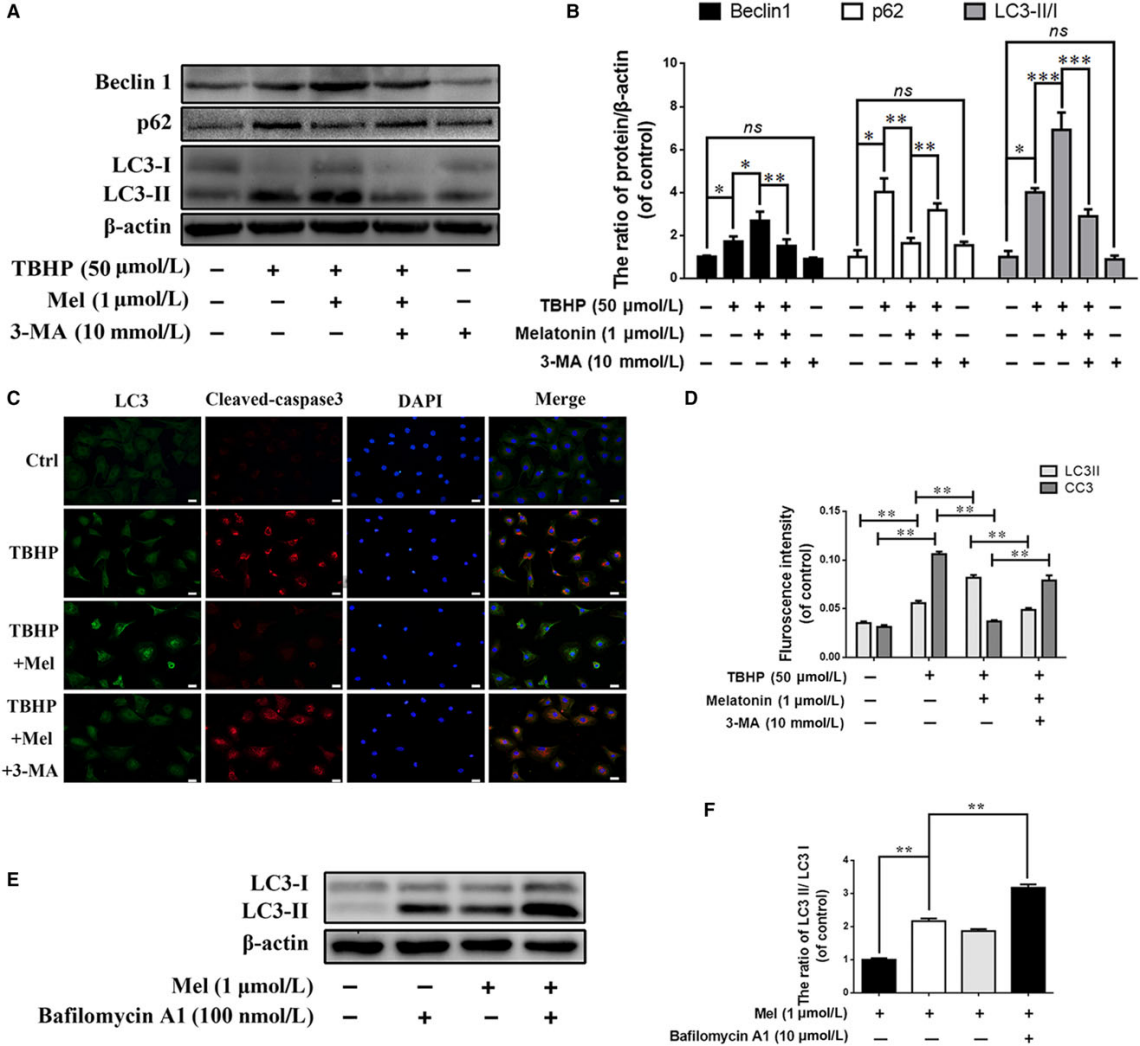
Effects of 3‐MA induced autophagy inhibition on the function of melatonin in endplate chondrocytes. (A) The protein expression of LC3, Beclin‐1 and p62 were evaluated by western blot. (B) Quantification of Beclin1, p62, LC3‐II/I immunoblots. (C, D) Double immunofluorescence of LC3 and cleaved‐caspase3 in Endplate chondrocytes (Green signal represents LC3, red signal represents cleaved‐caspase3, scale bar: 20 μm). Chondrocytes were treated with 100nM bafilomycin A1 for 1 hour. (E) The protein expression of LC3 in chondrocytes were evaluated by western blot. (F) Quantification of LC3 II/I immunoblots. The experiment was repeated at least three times, with a representative example shown; the values presented are the means ± SD of three independent experiments. Significant differences between groups are indicated as ***P < 0.001, **P < 0.01, *P < 0.05

To investigate whether melatonin protects EPCs against apoptosis via autophagy, the autophagy inhibitor 3‐MA and Atg5 siRNA was used in this study. The western blot results (Figure 3A) show that the LC3‐II/LC3‐I ratio and Beclin‐1 and p62 expression were similar to those in Figure 2C among the control, TBHP and TBHP + melatonin groups. However, in the 3‐MA group, Beclin‐1 expression and the LC3‐II/LC3‐I ratio were downregulated, and p62 expression was upregulated (*P* < 0.01), suggesting that 3‐MA indeed inhibits autophagy in EPCs, which was further confirmed by immunofluorescence staining for LC3 (Figure 3C). What is more, when Atg5 was knockdown by Atg5 siRNA, Beclin‐1 expression and the LC3‐II/LC3‐I ratio were downregulated, and p62 expression was upregulated (*P* < 0.01) (Figure S1C and D), indicating that atg5 siRNA suppress the activation of autophagy consistent with the previous study.^28^

**Figure 4:**
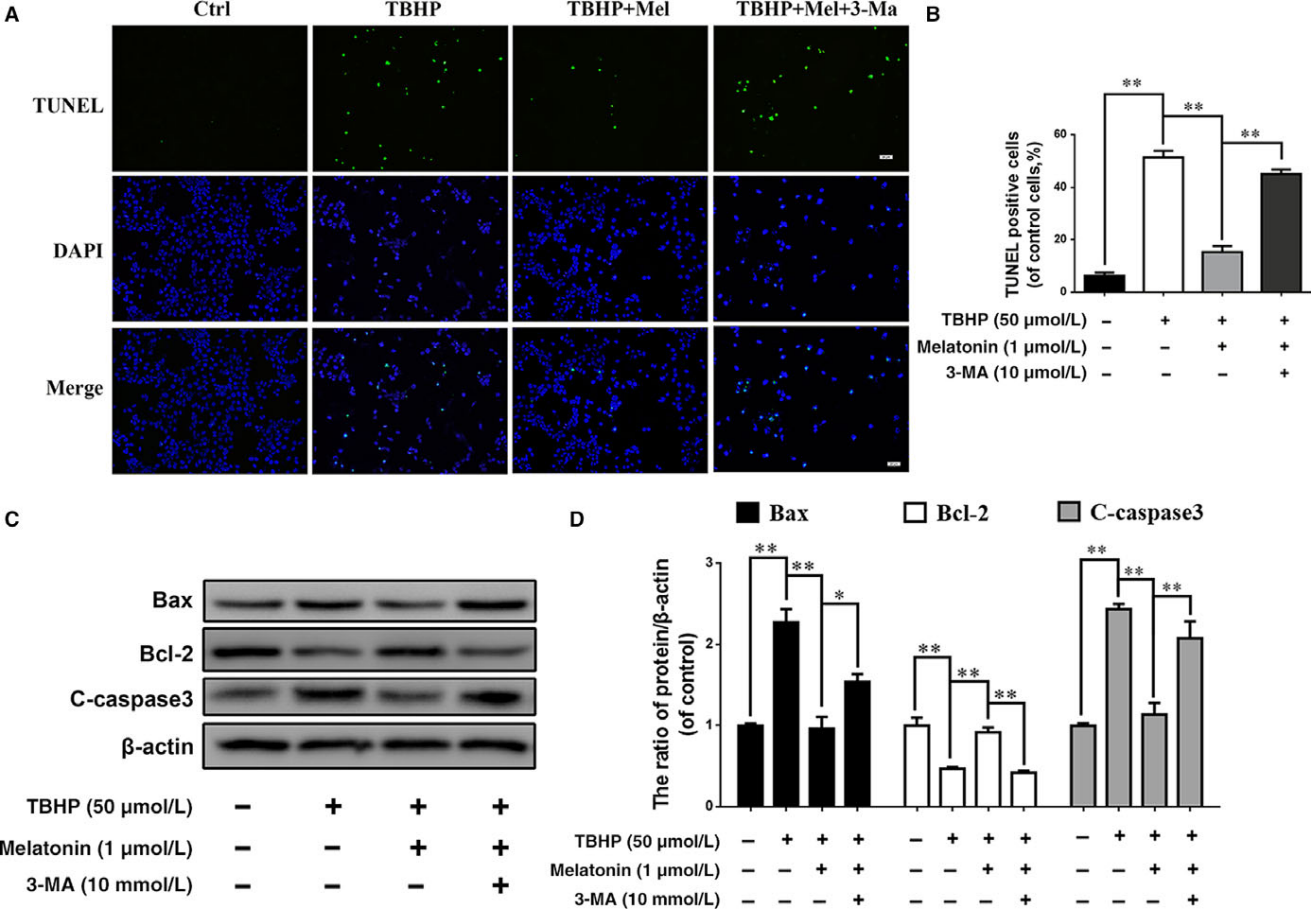
Melatonin treatment reduces oxidative stress induced apoptosis via promoting autophagy. Endplate chondrocytes were untreated (DMEM 10% FBS) or treated with TBHP (2 hours), with TBHP (2 hours) plus melatonin (4 hours), or with TBHP (2 hours) plus melatonin (4 hours) combined with 3‐MA (2 hours). (A) TUNEL assay was performed in chondrocytes (original magnification ×200, scale bar: 20 μm). (B) Quantification of number of apoptosis positive cells in each group. (C) The protein expression of cleaved‐caspase3, Bax and Bcl‐2 were evaluated by western blot. (D) Quantification of Bax, Bcl‐2, Cleaved‐caspase3 immunoblots. The experiment was repeated thrice, with a representative example shown; the values presented are the means ± SD of three independent experiments. Significant differences between groups are indicated as **P < 0.01, *P < 0.05

Meanwhile, in immunofluorescence staining of cleaved caspase‐3, the signal (red) was increased in the TBHP group and was decreased in the melatonin + TBHP group. However, the red signal was increased in the 3‐MA‐treated group compared with the melatonin + TBHP group, suggesting 3‐MA may eliminate the regulatory effect of melatonin on cleaved caspase‐3 in TBHP‐induced oxidative stress conditions. The TUNEL assay results revealed that the incidence of apoptosis was markedly increased in both TBHP‐treated and 3‐MA‐pretreated cells but was reduced in melatonin‐treated cells (Figure 4A; *P* < 0.01). In addition, western blot results revealed increased Bax and cleaved caspase‐3 expression and decreased Bcl‐2 expression in the TBHP group, while melatonin treatment reversed the expression pattern of these proteins. However, when 3‐MA was present, Bax, Cleaved caspase‐3 and Bcl‐2 expression returned to the levels of the TBHP group (*P* < 0.01). In addition, the expression of Cleaved‐caspase3 and Bax was remarkedly increased and Bcl‐2 was significantly decreased in EPCs when atg5 (key autophagy gene) was inhibited (Figure S1G and H). These data suggest that melatonin protect chondrocytes against apoptosis via autophagy activation. And the different expression level of LC3‐II was estimated in chondrocytes which are cultivated with bafilomycin A1 (a typical lysosomal inhibitor) and those without, is used to assess the activity of autophagic flux. As shown in Figure 3E, when EPCs were treated with bafilomycin A1, intracellular autophagosomes were inhibited and LC3 II expression was significantly increased compared with untreated group. Meanwhile, the content of LC3 II was further increased after Bafilomycin A1 treatment comparing to the group which treated with melatonin alone. These results suggest that melatonin increases the synthesis and clearance the autophagosomes in EPCs.

These results suggest that melatonin protects EPCs against oxidative stress‐induced apoptosis via stimulating autophagy.

### 3.4 Protective effect of melatonin against calcification in EPCs is through autophagy stimulation

**Figure 5:**
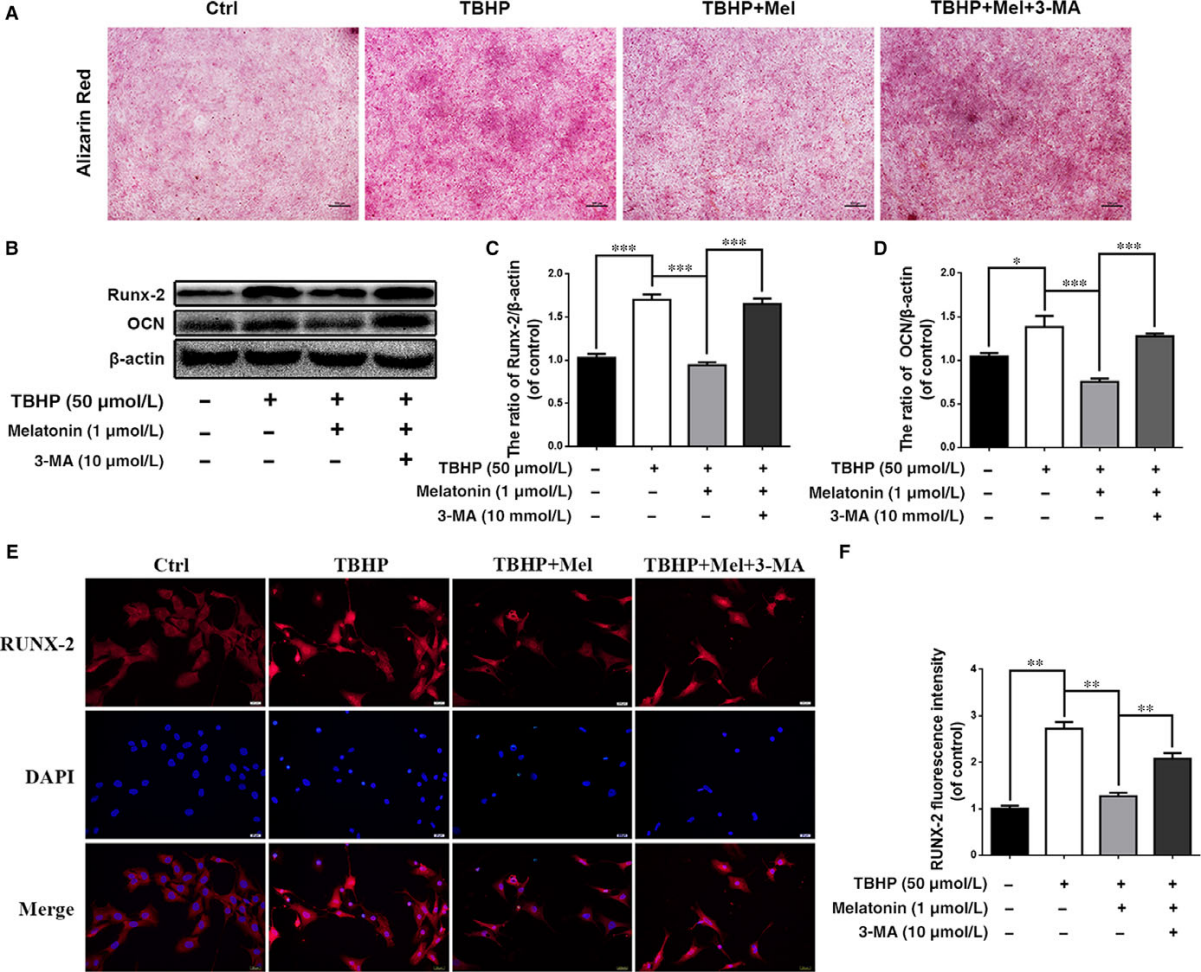
Melatonin protects endplate chondrocytes against oxidative stress induced calcification via promoting autophagy. Endplate chondrocytes were untreated (DMEM 10% FBS) or treated with TBHP (2 hours), with TBHP (2 hours) plus melatonin (4 hours), or with TBHP (2 hours) plus melatonin (4 hours) combined with 3‐MA (2 hours). (A) Endplate chondrocytes were treated with melatonin for 4 hours prior to TBHP for 2 hours, then cells were cultured in routine DMEM medium (without melatonin or TBHP) for 6 days, then cells were harvested and applied for Alizarin Red staining and imaged by phase‐contrast microscopy (magnification ×100, scale bar: 50 μm). (B) The protein expression of Runx‐2 and OCN in endplate chondrocytes were detected by western blot. (C, D) Quantification of Runx‐2 and OCN immunoblots. (E, F). Immunofluorescence of Runx‐2 protein in endplate chondrocytes. (Red signal represents Runx‐2, scale bar: 20 μm). The values are presented are the means ± SD of three independent experiments. Significant differences between groups are indicated as ***P < 0.001, **P < 0.01, *P < 0.05

Next, we investigated whether the protective effect of melatonin against calcification in EPCs is through autophagy stimulation. As shown in Figure 5A, alizarin red staining revealed that the amount of calcified nodules (red) was increased in TBHP‐treated cells and decreased after melatonin treatment. In addition, the melatonin‐induced decrease in calcified nodules was eliminated when 3‐MA was present (Figure 5A). The western blot results revealed that the expression of calcification‐related proteins, such as Runx‐2 and OCN, was increased with the TBHP treatment. Melatonin treatment decreased the expression of calcification‐related proteins (Runx‐2 and OCN), but this effect was reversed by the 3‐MA treatment (Figure 5 B‐D; *P* < 0.01). Furthermore, we used immunofluorescence to assess Runx‐2 translocation. The results revealed that Runx‐2 nuclear localization was increased in the TBHP group; melatonin treatment partially decreased the nuclear localization of Runx‐2. However, the autophagy inhibitor 3‐MA reversed this phenomenon (Figure 5E and F; *P* < 0.01). In addition, the expression of Runx‐2 and OCN was remarkedly increased in EPCs when atg5 (key autophagy gene) was inhibited (Figure S1E and F). These data suggest that melatonin protect chondrocytes against calcification via autophagy activation.

These results indicate that the protective effect of melatonin against calcification in EPCs is through autophagy stimulation.

### 3.5 Melatonin protects EPCs against apoptosis through Sirt1‐mediated autophagy stimulation

**Figure 6:**
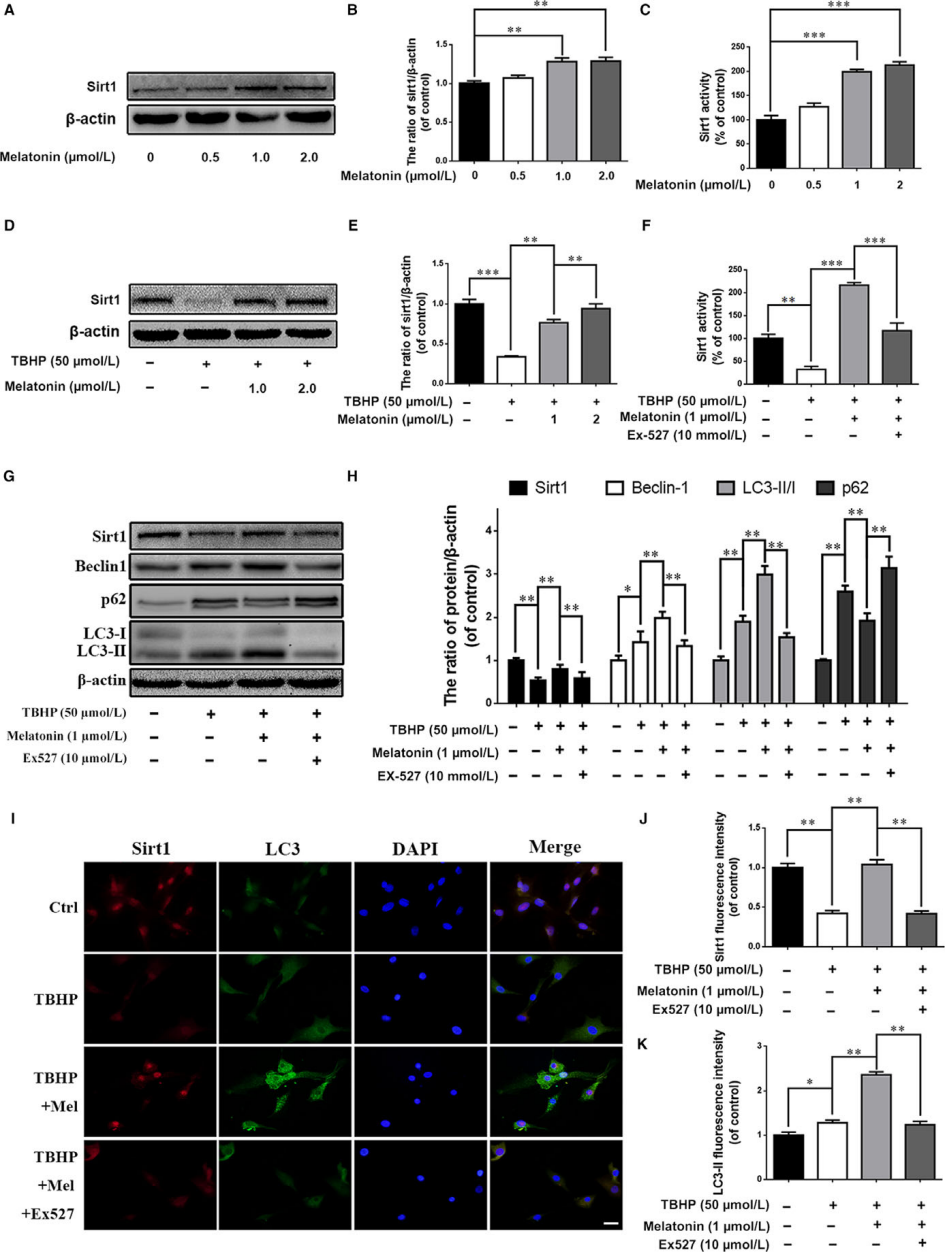
Sirt1 inhibition significantly attenuated melatonin‐induced autophagy in endplate chondrocytes. (A) Endplate chondrocytes were incubated with various concentrations of melatonin for 24 hours, and the expression of Sirt1 was determined by western blotting. (B) Quantification of Sirt1 immunoblots. (C) Endplate chondrocytes were incubated with various concentrations of melatonin for 24 hours. Sirt1 activity in endplate chondrocytes was determined by a Sirt1 assay kit. (D) Endplate chondrocytes were incubated with various concentrations of melatonin prior to TBHP treatment (50 μM), and Sirt1 expression was determined by western blotting. (E) Quantification of Sirt1 immunoblot. Endplate chondrocytes were treated differently. Endplate chondrocytes were untreated (DMEM 10% FBS) or treated with TBHP (2 hours), with TBHP (2 hours) plus melatonin (4 hours), or with TBHP (2 hours) plus melatonin (4 hours) combined with EX‐527 (2 hours). (F) After the cells were subjected to the above treatment, Sirt1 activity in endplate chondrocytes was determined by a Sirt1 assay kit. (G) The protein expression of Sirt1, LC3, Beclin‐1 and p62 in the endplate chondrocytes was detected by western blotting. (H) Quantification of Sirt1, LC3‐II/I, p62, and Beclin‐1 immunoblots. (I‐K) Immunofluorescence double‐label staining of Sirt1 and LC3 protein was observed in endplate chondrocytes. (Green signal represents LC3‐II, red signal represents Sirt1, scale bar: 20 μm). The experiment was repeated thrice, and a representative example is shown. The values are presented are the means ± SD of three independent experiments. Significant differences between groups are indicated as *** *P* < 0.001, **P < 0.01, *P < 0.05 and ns *P* > 0.05

Sirt1 has been reported to be the upstream regulator of autophagy.^29^ Intriguingly, we found that melatonin treatment increases Sirt1 expression in a dose‐dependent manner (Figure 6A and B). Meanwhile, using the Sirt1 activity assay kit, we measured Sirt1 activity following melatonin treatment, and the results revealed that melatonin upregulates Sirt1 activity (Figure 6C). Additionally, we found that melatonin may promote Sirt1 expression as well as activity under conditions of TBHP‐induced oxidative stress (Figure 6D‐F). Next, we investigated the relationship between Sirt1 and autophagy, as well as apoptosis and calcification, in EPCs.

EX‐527 was used as an Sirt1 inhibitor in our study and revealed that Sirt1 expression and activity were inhibited by EX‐527, suggesting that EX‐527 may indeed suppress Sirt1 activity (Figure 6F). We found that EX‐527 treatment may suppress melatonin‐induced autophagy, as indicated by the decreased LC3‐II/LC3‐I ratio and Beclin‐1 expression and increased p62 expression in the Ex 527 group compared with the TBHP + melatonin group (Figure 6G and H; *P* < 0.01). Immunofluorescence staining of Sirt1 and LC3 also confirmed these findings (Figure 6I‐K; *P* < 0.01).

**Figure 7:**
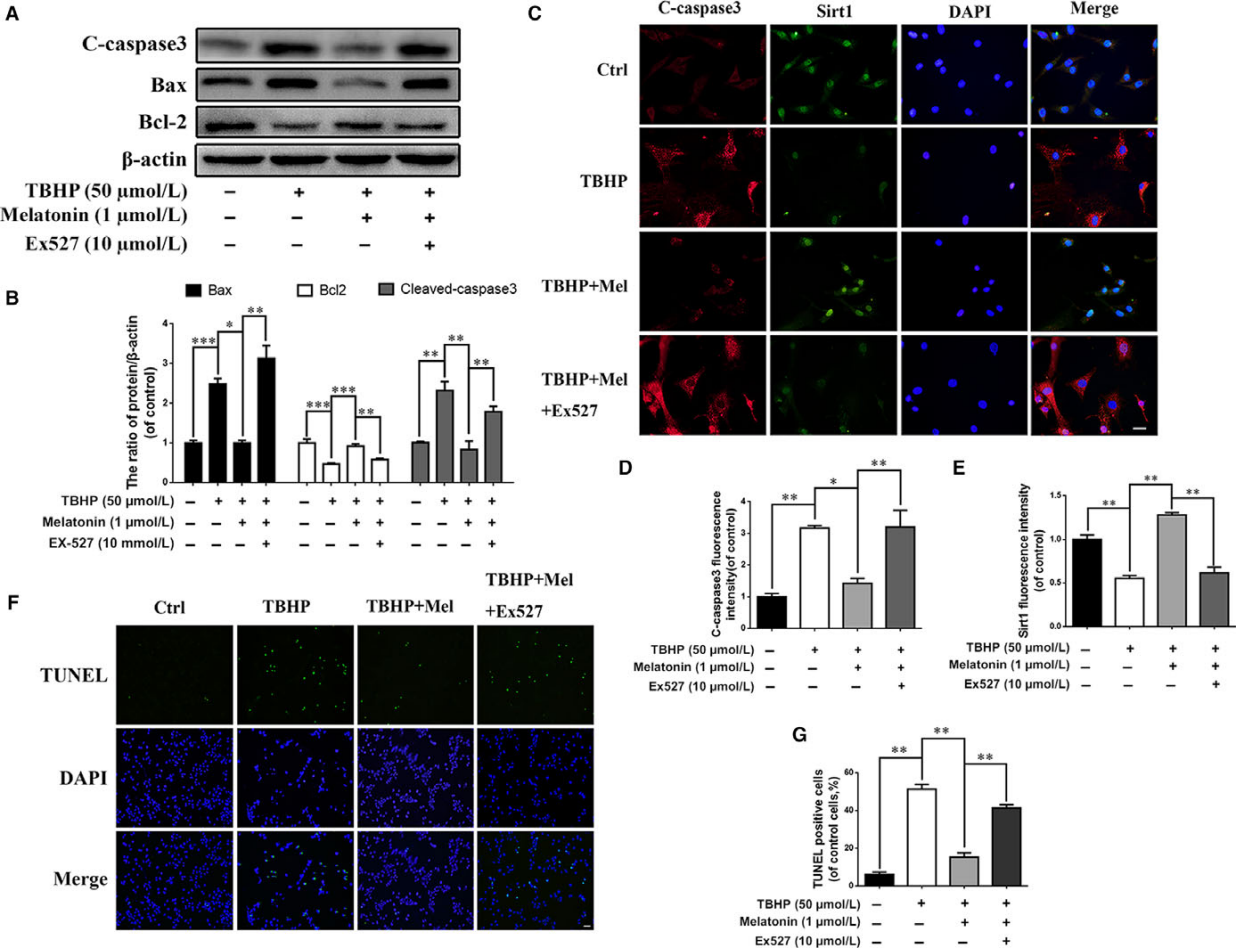
The protective effect of melatonin against apoptosis is associated with Sirt1‐autophagy signaling pathway. Endplate chondrocytes were untreated (DMEM 10% FBS) or treated with TBHP (2 hours), with TBHP (2 hours) plus melatonin (4 hours), or with TBHP (2 hours) plus melatonin (4 hours) combined with EX‐527 (2 hours). (A) The expression of cleaved‐caspase3, Bax and Bcl‐2 were determined by western blot in endplate chondrocytes. (B) Quantification of Cleaved‐caspase3, Bax, Bcl‐2 immunoblots. (C‐E) Immunofluorescence double label staining of Sirt1 and cleaved‐caspase3 in endplate chondrocytes were observed by Olympus fluorescence microscope. (Green signal represents Sirt1, red signal represents cleaved‐caspase3, scale bar: 20 μm). (F) TUNEL assay was performed in endplate chondrocytes as treated above (original magnification ×200, scale bar: 20 μm). (G) Quantification of apoptosis positive cells numbers in each group. The experiment was repeated thrice, with a representative example shown; the values are presented are the means ± SD of three independent experiments. Significant differences between groups are indicated as ***P < 0.001, **P < 0.01, *P < 0.05

Next, we assessed how EX‐527‐induced Sirt1 suppression affects apoptosis in EPCs. As shown in the western blot results, Bax and cleaved caspase‐3 expression was decreased, while Bcl‐2 expression was increased in the melatonin group. However, Bax and cleaved caspase‐3 expression was increased, and Bcl‐2 expression was decreased in the presence of EX‐527 (Figure 7A and B; *P* < 0.01), indicating that EX‐527‐induced Sirt1 suppression may attenuate the inhibitory effect of melatonin on apoptosis. This phenomenon was further confirmed by immunofluorescence staining of cleaved caspase‐3 and TUNEL assays (Figure 7C‐F; *P* < 0.01).

Together, these results suggest that melatonin protects EPCs against apoptosis by stimulating Sirt1‐mediated autophagy.

### 3.6 Melatonin protects EPCs against calcification by stimulating Sirt1‐mediated autophagy

**Figure 8:**
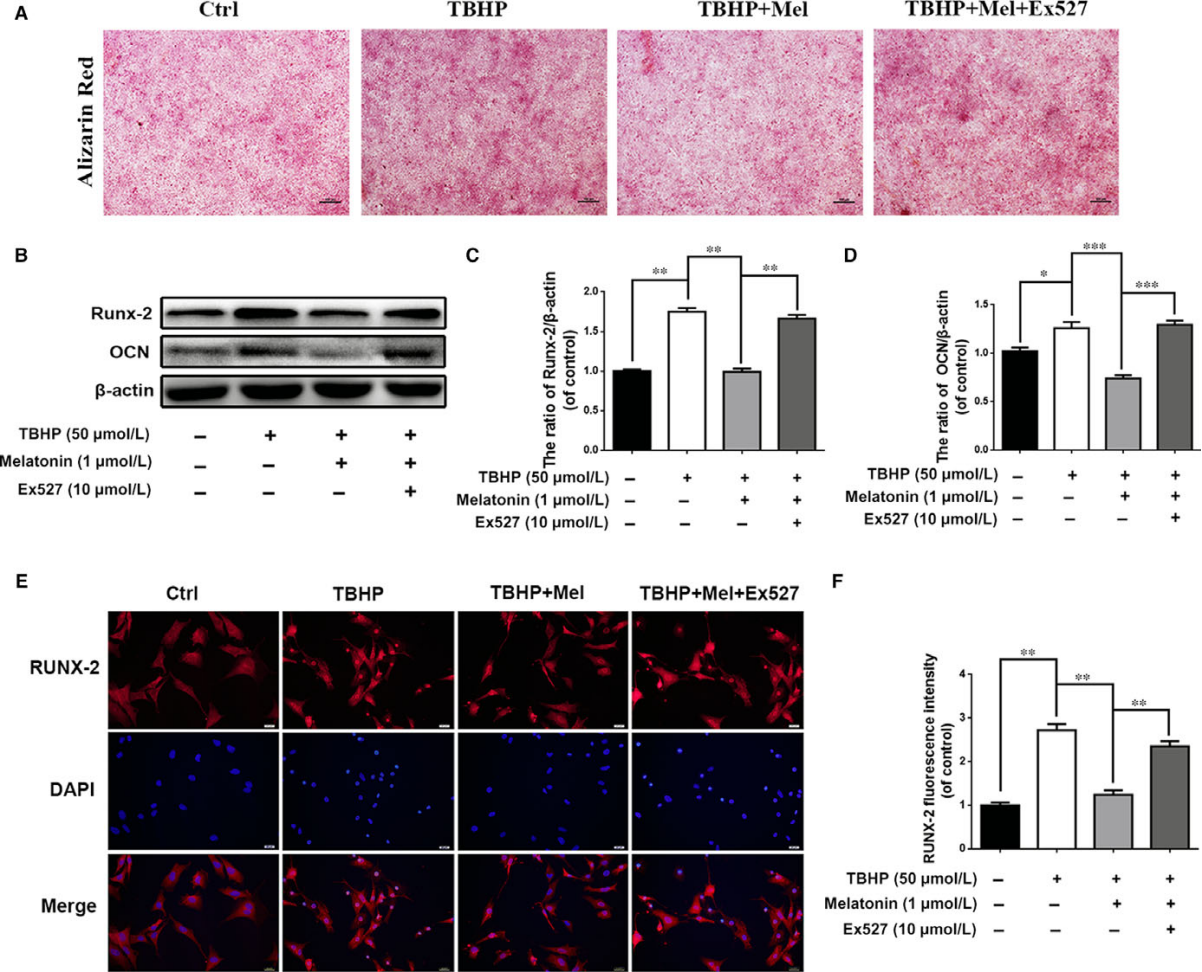
The protective effect of melatonin against calcification is associated with Sirt1‐autophagy signaling pathway. (A) Alizarin Red staining of endplate chondrocytes: Endplate chondrocytes were treated differently. Endplate chondrocytes were untreated (DMEM 10% FBS) or treated with TBHP (2 hours), with TBHP (2 hours) plus melatonin (4 hours), or with TBHP (2 hours) plus melatonin (4 hours) combined with EX‐527 (2 hours), then cells were changed to normal media for 6 days. (B) The expression of Runx‐2 and OCN in endplate chondrocytes were determined by western blot. (C, D) Quantification of immunoblots of OCN and Runx‐2. (E, F) Immunofluorescence of Runx‐2 protein in endplate chondrocytes. (Red signal represents Runx‐2, scale bar: 20 μm) The experiment was repeated three times, with a representative example shown; The values are presented are the means ± SD of three independent experiments. Significant differences between groups are indicated as ***P < 0.001, **P < 0.01, *P < 0.05

To determine whether melatonin protects EPCs against calcification through Sirt1‐mediated autophagy stimulation, we again used EX‐527 in our study. As shown in the alizarin red staining results, EX‐527‐induced Sirt1 suppression substantially inhibited the regulatory effect of melatonin on EPC calcification (Figure 8A).

The western blot results revealed that the TBHP‐induced increase in Runx‐2 and OCN expression was decreased when cells were treated with melatonin (Figure 8B‐D; *P* < 0.01); however, the expression level of these two proteins almost returned to the level of the TBHP treated group when EX‐527 was present. Additionally, we checked the nuclear localization of Runx‐2 in EPCs. The results demonstrated that TBHP may drive Runx‐2 nuclear transportation, while melatonin may prevent this translocation. However, when EX‐527 was present, this melatonin‐induced prevention was reduced (Figure 8E and F).

These results suggest that melatonin may protect EPCs against calcification through the stimulation of Sirt1‐mediated autophagy.

### 3.7 Melatonin ameliorates the IDD process in a puncture‐induced rat model

**Figure 9:**
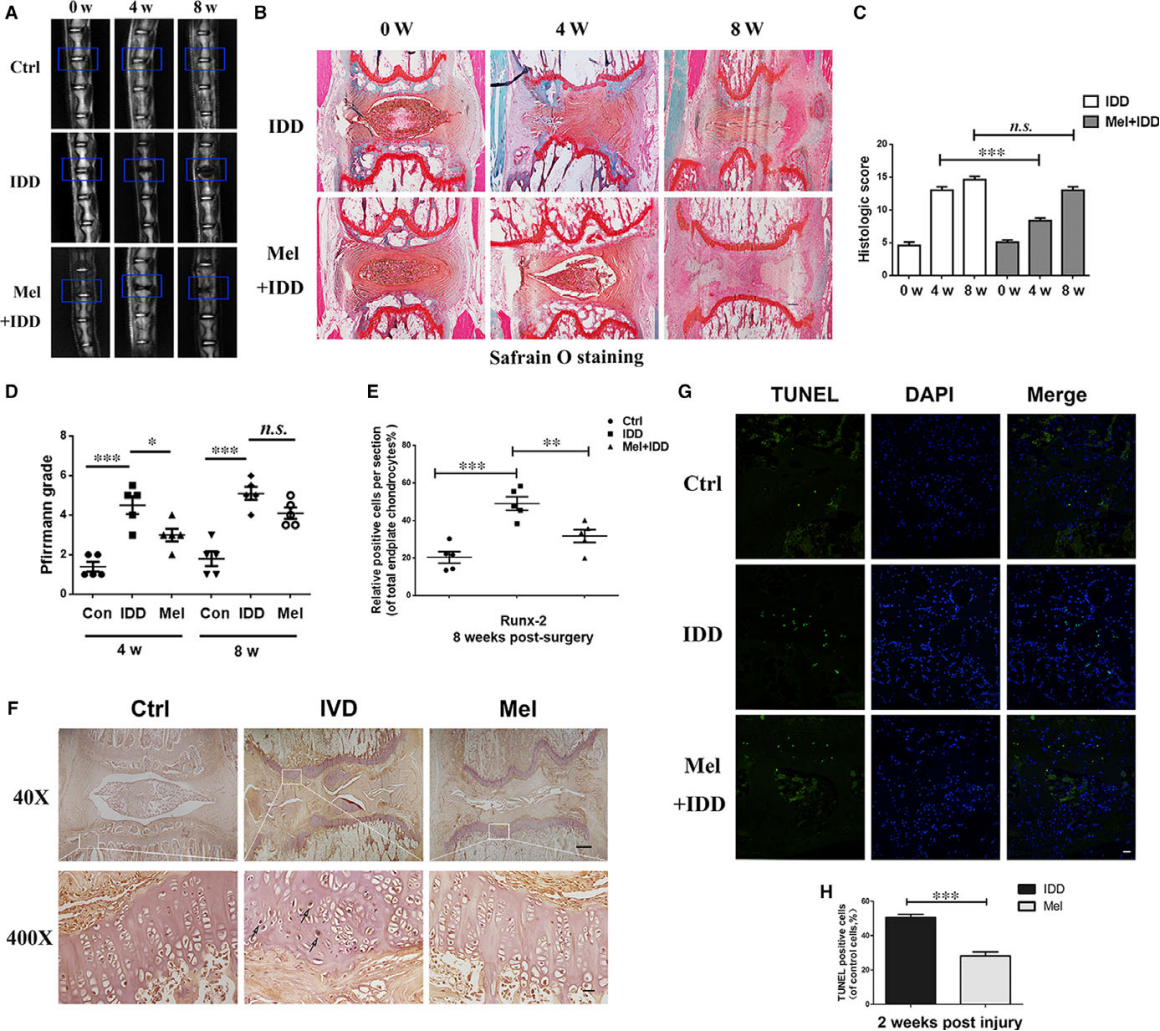
Melatonin treatment ameliorates rat IDD in vivo. The rat IDD model was established by stabbing the whole annulus fibrosus (AF) layer through the tail skin using needles (27G) for 1 minute. At 0, 4, and 8 weeks, degenerated discs were evaluated with MRI or safranin O staining (SO; original magnification 40×, scale bar: 50 μm). (A) T2‐weighted MRI images of a rat tail with a needle‐punctured disc at 0, 4 and 8 weeks post‐surgery (blue boxes: location of the needle‐puncture disc). (B) Representative SO staining of disc samples from different experimental groups at 4 and 8 weeks post‐surgery (original magnification 40×, scale bar: 100 μm). Three sections were randomly selected for quantification, and a representative example is shown. (C) The histological grades were evaluated at 4 and 8 weeks post‐surgery in the three groups (6 rats per group). (D) The Pfirrmann MRI scores in the three groups at weeks 4 and 8 (6 rats at each time point for each group). (E, F) Immunohistochemical staining of Runx‐2 expression in the endplate cartilage samples (original magnification 40× scale bar: 100 μm; original magnification 400×, scale bar: 20 μm), and the number of positive cells was counted by three double‐blinded researchers using Image Pro‐Plus. (G, H) TUNEL assays in endplate cartilage samples and positive chondrocytes cells were counted by three double‐blinded researchers using Image Pro‐Plus (original magnification ×200, scale bar: 20 μm). Values are displayed as means ± SD of 6 rats per group. Significant differences between groups are indicated as ***P < 0.001, **P < 0.01, *P < 0.05

We established a puncture‐induced IDD model in rats to evaluate the effects of melatonin on IDD in vivo. The IDD degeneration level was assessed by MRI in rats and was quantified by Pfirrmann MRI grading. MR images obtained at 4 weeks after the puncture revealed T2‐weighted signal intensities were stronger in the melatonin‐treated group than in the saline group; similar results were also observed at 8 weeks (Figure 9A). In addition, the Pfirrmann MRI grade scores, which indicate the degree of disc degeneration, were significantly lower in the melatonin‐treated rats than in the IDD rats at 4 (*P* < 0.05) and 8 weeks (*P* < 0.05; Figure 9D).

S‐O stains proteoglycans and glycosaminoglycans. As shown in Figure 9B, we found that the CEP was thicker and that the structure was more intact in the melatonin‐treated group than in the vehicle group, indicating that melatonin has beneficial effects for protecting endplate cartilage. Intriguingly, the results also revealed that not only the structure but also the extracellular matrix of nucleus pulposus tissues was better preserved in the melatonin‐treated group, implying that melatonin may also be beneficial for nucleus pulposus tissues. For SO staining at the 8‐week time point, the effect of melatonin on morphological and extracellular matrix preservation of nucleus pulposus was much weaker than at the 4‐week time point, but differences between the endplate cartilage of the melatonin‐treated and vehicle groups was still present: the endplate cartilage was better preserved in the melatonin‐treated group than in the vehicle group, suggesting that long‐term melatonin treatment (8 weeks) may ameliorate IDD.

We also assessed endplate cartilage calcification and apoptosis in melatonin‐treated rats. The immunohistological staining results revealed that the number of Runx‐2‐positive cells was reduced in the melatonin group compared with the IDD group (Figure 9E and F), indicating that calcification was decreased in the melatonin‐treated rats. We also used TUNEL assays to stain apoptotic endplate cartilage cells. The results revealed that the number of TUNEL‐positive cells (green) was decreased in the melatonin group compared with the IDD group (Figure 9G and H), suggesting that apoptosis was reduced in the endplate cartilage of melatonin‐treated rats.

This in vivo study revealed that melatonin treatment ameliorates the IDD process; meanwhile, melatonin may suppress endplate cartilage calcification and apoptosis.

## 4 DISCUSSION

Although melatonin has proven beneficial effects in intervertebral discs under physiological and pathological conditions, its effect and mechanism of action on EPCs are poorly understood. The main finding of this study is that in primary EPCs, melatonin may promote autophagy, which subsequently inhibits apoptosis and calcification‐related protein and gene expression via the Sirt1 signalling pathway under oxidative stress conditions.

In vivo and in vitro evidence has shown that oxidation and oxidation products are widely present in degenerated intervertebral discs.^30^, ^31^, ^32^ Studies have reported that oxidative stress may increase the incidence of apoptosis through the mitochondria‐dependent pathway in various cell types^33^; in addition, reactive oxygen species and oxidative stress products have been shown to increase ectopic calcification formation in cardiovascular cells.^34^, ^35^ Han et al reported that oxidative stress may induce apoptosis and promote calcification in disc cartilage endplate cells.^7^ These studies suggest that oxidative stress is a common pathological factor for apoptosis and calcification in cells, including EPCs. Therefore, we tested the mechanism of action of melatonin on EPC apoptosis and calcification in a TBHP‐induced system of oxidative stress.

Oxidative stress may induce mitochondrial dysfunction through increasing apoptosis‐related proteins, such as Bax, and decreasing anti‐apoptosis‐related proteins, such as Bcl‐2, which ultimately activates the caspase protein family and leads to chondrocyte apoptosis.^36^ In the present study, we found that melatonin upregulates Bcl‐2 expression while downregulating Bax and cleaved caspase‐3 expression, suggesting that melatonin may protect EPCs against oxidative stress‐induced apoptosis. Alizarin red staining, which is a reliable marker of calcification,^37^ showed that melatonin can reverse the calcification induced by oxidative stress. Runx‐2 and OCN expression levels were both decreased after melatonin treatment under oxidative stress conditions, which also suggests that melatonin can attenuate EPC calcification. Together, these results demonstrate that melatonin not only suppresses apoptosis but also reduces calcification in EPCs under oxidative stress; however, how melatonin protects EPCs against apoptosis and calcification remains unclear.

In recent studies, autophagy has been thought to be a protective mechanism under pathological conditions, degrading damaged organelles or proteins.^38^ Autophagy has been proven by our group^25^ as well as other groups^39^, ^40^, ^41^, ^42^ to postpone IDD processes in vitro and in vivo. Xu et al reported that rapamycin‐stimulated autophagy may inhibit intermittent mechanical stress‐induced calcification in cartilage EPCs.^43^, ^44^ Chen et al demonstrated that oxidative stress induces autophagy in EPCs, while the inhibition of autophagy by bafilomycin A1 may increase apoptosis.^45^ The studies above suggest that autophagy may be a protective mechanism against apoptosis and calcification in EPCs.

Melatonin has been documented to regulate autophagy in various cell types^46^, ^47^; however, its effect on EPCs was not uniform. Melatonin was reported to promote autophagy in diabetic cardiomyopathy,^48^ peripheral neuropathy,^49^ and to suppress autophagy in type 2 diabetic osteoporosis.^50^ However, how melatonin modulates autophagy in EPCs is still unclear: studies in articular chondrocytes have not shown that melatonin may regulate autophagy. Therefore, we were curious to see how melatonin may regulate autophagy in EPCs.

LC3 and Beclin‐1 are two key essential proteins for autophagy initiation, and p62 is indispensable for autophagic degradation.^51^, ^52^ We used these proteins as markers to evaluate melatonin‐induced autophagy. In our study, we found that LC3 and Beclin‐1 expression was increased with the melatonin treatment, indicating that autophagy initiation was activated; meanwhile, p62 was decreased after melatonin treatment, suggesting that autophagic degradation was also promoted (Figure 2A). Therefore, we hypothesize that melatonin may protect EPCs against apoptosis and calcification via promoting autophagy.

To investigate the association between autophagy and apoptosis or calcification in EPCs, the classical inhibitor of autophagolysosome formation 3‐MA was used in this study. With 3‐MA treatment, melatonin‐induced autophagy was downregulated, and autophagic flux was obstructed, which was indicated by increased p62 expression (Figure 3A). Meanwhile, the protective effects of melatonin on EPCs were eliminated, as shown by the increased incidence of apoptosis and calcification (Figures 4A and 5A). Additionally, the expression of apoptosis indicators, such as cleaved caspase‐3, and of calcification indicators, such as Runx‐2 and OCN, was increased with the combined 3‐MA pretreatment and melatonin treatment under oxidative stress (Figures 4 and 5). Our results suggest that melatonin‐induced autophagy is beneficial against apoptosis and calcification in EPCs. Actually, autophagic flux is often defined as a measure of autophagic degradation activity and previous study suggest that disruption of autophagic flux contribute to cell death in many degenerative diseases.^53^ Consistent with our previous study,^25^ although TBHP promotes autophagy, autophagic flux was destroyed in EPCs. And the conversion of LC3 I/II in EPCs indicate that melatonin could activated autophagy and resumed autophagy flux in chondrocytes. Thus, melatonin protect chondrocytes against apoptosis and calcification via autophagy. Next, we wondered how melatonin promotes autophagy in EPCs.

Sirt1, an NAD+‐dependent deacetylase, is reportedly decreased with age during IDD progression.^54^ In addition, recent studies have reported that Sirt1 protects nucleus pulposus cells against apoptosis and senescence in vitro.^55^, ^56^ Studies have also shown that Sirt1 promotes autophagolysosome formation, which may further suppress apoptosis and senescence in the brain and other tissues.^57^, ^58^, ^59^ In our study, we found that Sirt1 expression and activity was increased with melatonin treatment (Figure 6A‐E), suggesting that Sirt1 may play a role in the regulatory effect of melatonin on autophagy in EPCs.

To determine whether melatonin promotes autophagy through Sirt1, EX‐527, an inhibitor of Sirt1, was used in our study. The results revealed that melatonin activates autophagy under oxidative stress conditions. However, the effect was weakened when cells were pretreated with EX‐527, suggesting the melatonin modulates autophagy through stimulating the Sirt1 pathway (Figure 6G and H). The incidence of apoptosis was increased with EX‐527 treatment, according to the western blot results, immunofluorescence and TUNEL assays in EPCs (Figure 7). Additionally, the effect of melatonin on eliminating EPC calcification was inhibited by EX‐527 treatment (Figure 8). These results suggest that the effect of melatonin on reducing apoptosis and calcification in EPCs was through the Sirt1‐autophagy signalling pathway.

Intriguingly, aside from endplate cartilage, the nucleus pulposus was also better preserved in the melatonin‐treated group than in the IDD untreated group in the in vivo study, suggesting that melatonin may contribute to IDD therapeutics also by targeting nucleus pulposus (Figure 9). A recent study revealed that melatonin can inhibit nucleus pulposus cell proliferation and extracellular matrix remodelling in nucleus pulposus cells,^20^ but this study was carried out with only in vitro experiments. Our in vivo results may support this study, showing that melatonin treatment may benefit IDD through targeting EPCs and through regulating nucleus pulposus cells.

**Figure 10:**
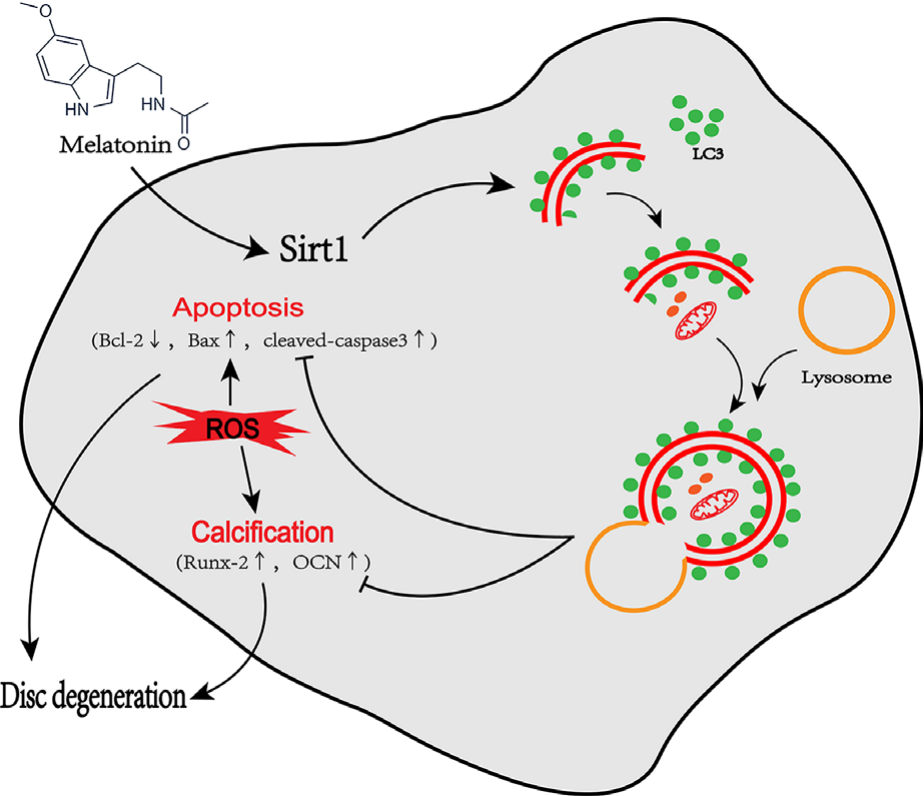
Proposed working model. As one of the main contributors to intervertebral disc degeneration (IDD), ROS may lead to apoptosis and calcification in endplate chondrocytes (EPCs). Melatonin may promote Sirt1 expression and activity, thereby activating autophagy in EPCs. Activated autophagy may suppress the expression of apoptotic proteins, such as Bax and cleaved caspase‐3, promote anti‐apoptotic protein Bcl‐2 expression and inhibit apoptosis in EPCs. Autophagy activated by melatonin may also downregulate ROS‐induced Runx‐2 and OCN expression by suppressing EPC calcification

In conclusion, this study provides evidence that melatonin may have anti‐apoptosis and anti‐calcification effects in EPCs, and the associated mechanism of action may be related to the Sirt1‐autophagy signalling pathway. The detail molecular mechanisms of melatonin in EPCs on IDD are illustrated in Figure 10. Our observations provide a further explanation for the association between pinealectomy surgery and IDD.^17^, ^20^ And our efforts suggest that melatonin may be a new, potentially effective medicine for IDD prevention and treatment.
