# Supplementary figures and images for: Melatonin protects vertebral endplate chondrocytes against apoptosis and calcification via the Sirt1‐autophagy pathway
Source: J Cell Mol Med. 2018 Oct 24;23(1):177–93. doi: 10.1111/jcmm.13903 (PMC6307776; doi:10.1111/jcmm.13903)

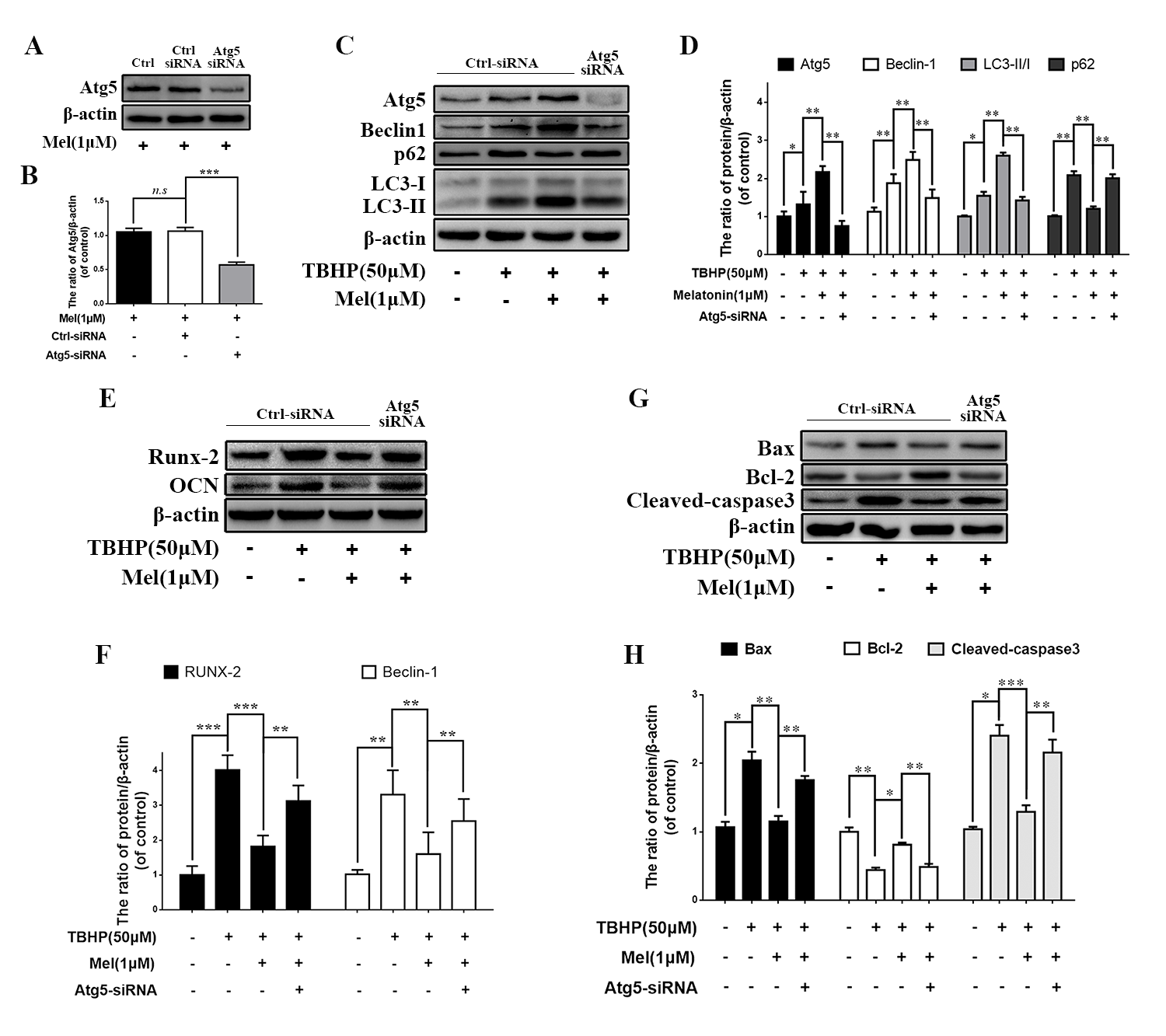

Supplement: Supplementary file 1 [file JCMM-23-177-s001.tif]
